# Direct arylation catalysis with chloro[8-(dimesitylboryl)quinoline-κN]copper(I)

**DOI:** 10.3762/bjoc.12.272

**Published:** 2016-12-15

**Authors:** Sem Raj Tamang, James D Hoefelmeyer

**Affiliations:** 1 Department of Chemistry, University of South Dakota, 414 E. Clark St., Vermillion, SD 57069, USA

**Keywords:** catalysis, C–C coupling, C–H activation, copper, direct arylation

## Abstract

We report direct arylation of arylhalides with unactivated sp^2^ C–H bonds in benzene and naphthalene using a copper(I) catalyst featuring an ambiphilic ligand, (quinolin-8-yl)dimesitylborane. Direct arylation could be achieved with 0.2 mol % catalyst and 3 equivalents of base (KO(*t*-Bu)) at 80 °C to afford TON ≈160–190 over 40 hours.

## Introduction

Coupling of aryl C–C bonds is invaluable in organic synthesis, and has been the subject of much research with the aim to lower cost and improve atom efficiency [1–5]. The earliest and most developed methods involve the reaction of C–M (M = Li, B, Mg, Si, Sn, Zn) with C–X (X = halide, triflate, tosylate, or aryliodide (as Ar_2_I^+^)) using Pd-based catalysts. These methods achieve coupling of aryl C–C bonds with high activity and selectivity; however, the reactants are quite expensive and there is significant opportunity to improve the atom efficiency. Additionally, there is impetus to utilize less expensive metal catalysts or even metal-free reactions. Recently, there has been rapid progress toward the use of C–H bonds as a reactive functional group [6]. While it would be highly desirable to utilize C–H/C–H coupling reactions [7], low reactivity and selectivity are significant obstacles. The use of directing groups can improve the selectivity, but could contribute additional reaction steps and expense. Therefore, C–X/C–H (X = halogen) direct arylations are an important avenue of investigation that may represent a balance between cost, activity, and selectivity [8–13].

The C–X/C–H direct arylation reaction can be achieved with X = halogen, triflate, tosylate [14], B(OH)_2_ [15–20], SnR_3_ [21], Si(OR)_3_ [22], or with the use of aryliodonium salts [23]; wherein halogen atoms represent the most cost-effective and atom-efficient functional group. The reactions typically utilize expensive transition metal catalysts (Pd, Rh, Ir) at high loadings (typically 10 mol %), and require a large amount of strong base (typically 3 equivalents KO(*t*-Bu)). There has been some success using inexpensive first-row transition metals (Fe [24–25], Co [26–28], Ni [29–33], and Cu [34–37]) or an aluminum-based metal-organic framework [38], and there are several reports of metal-free direct arylation reactions in which the reaction is promoted with 2–3 equivalents KO(*t*-Bu) and (typically) 10–30 mol % of an additive [39–48].

**Scheme 1:**
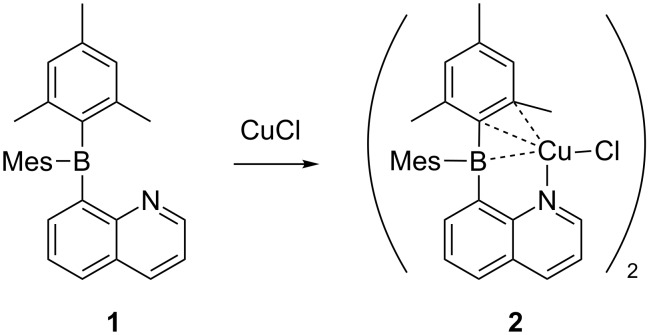
Coordination of Cu(I) with the ambiphilic ligand **1** to form the catalyst **2**.

The use of ambiphilic molecules as ligands for transition metals has given rise to an important new class of catalysts [49]. In previous work from our laboratory, we prepared the intramolecular frustrated Lewis pair 8-quinolyldimesitylborane (**1**) and its complexes with Cu(I), Ag(I), and Pd(II) [50]. Recently, we reported the Pd(II) complex catalyzed Heck-type C–C coupling [51]. The observation may implicate reductive elimination and oxidative addition can cycle repeatedly on the palladium center coordinated to **1**. With this in mind, we sought to utilize the Cu(I) complex, chloro[8-(dimesitylboryl)quinoline-κN]copper(I) (**2**, Scheme 1), and investigate its performance as a catalyst for direct arylation reactions. We note that compound **2** consists of the ambiphilic ligand **1** coordinated to Cu(I) via nitrogen atom coordination and an η^3^-BCC interaction [52]. Herein we report the use of the preformed catalyst **2** and demonstrate good activity for C–X/C–H direct arylation reactions.

## Results and Discussion

The results of direct arylation catalysis are summarized in Table 1. We performed the reaction of iodobenzene with benzene/DMF (10:1 v/v) at 80 °C using a catalyst loading of 2 mol % of **2** and 30 equivalents KO(*t*-Bu) (Table 1, entry 1). After 10 hours the yield of biphenyl was 94% according to GC–MS analysis. A control reaction (Table 1, entry 4) of iodobenzene with benzene/DMF (10:1 v/v) using 2 mol % Cu(I)Br and 30 equivalents KO(*t*-Bu) gave 5% yield of biphenyl after 10 hours. Reactions performed in the absence of catalyst, using 3 or 30 equivalents KO(*t*-Bu) gave no reaction (Table 1, entries 5 and 6). While the high yield of the direct arylation suggests that the product cannot be exclusively obtained from homocoupling of iodobenzene, we performed additional experiments to demonstrate a heterocoupling pathway. The coupling of benzene with *p*-iodoanisole at 2 mol % loading of catalyst **2** was evaluated (Table 1, entry 2). We were pleased to find that the reaction yielded 4-methoxybiphenyl according to GC–MS analysis, and the isolated yield after flash column chromatography was 77%. Encouraged by these findings, we attempted the reaction with lower catalyst loadings and 3 equivalents KO(*t-*Bu). The reaction of iodobenzene with benzene with catalyst loading of 0.5 mol % gave biphenyl in isolated yield of 85% (Table 1, entry 9). The reaction of *p*-iodoanisole with benzene using 0.2 or 0.3 mol % of catalyst **2** gave 4-methoxybiphenyl in isolated yields of 33% and 53%, respectively, which indicate a TON ≈190 (Table 1, entries 11 and 17). The reaction of *p*-iodotoluene and benzene (Table 1, entries 10 and 13) with 3 equivalents KO(*t-*Bu) gave 4-methylbiphenyl in yields of 25% and 52% using 0.3 and 0.5 mol % of catalyst **2**, respectively (TON ≈170 for the latter). The reactions of *p*-iodophenol (Table 1, entry 14) or *p*-nitroiodobenzene (Table 1, entry 3) with benzene were very sluggish with only trace quantities (<1% yield) of products. The coupling of *m*-bromotoluene and *m*-bromochlorobenzene with benzene proceeded more slowly (Table 1, entries 15 and 16). The yields of 3-methylbiphenyl and 3-chlorobiphenyl at catalyst loading of 0.3 mol % and 3 equivalents KO(*t-*Bu) were 8% and 4%, respectively. In all of the reactions, we note that metallic precipitates did not form, and the solutions did not become dark.

**Table 1:** Coupling reaction of aryl halides with benzene. Reactions were carried out with 4 mL of benzene and 0.4 mL of DMF.

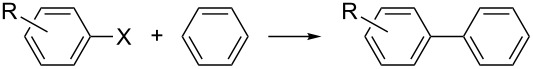							

entry	catalyst	cat. (mol %)	KO(*t-*Bu) (equiv)	X	R^a^	time (h)	yield (%)

1	2	2	30	I	H	10	94
2	2	2	30	I	*p*-OMe	20	77
3	2	2	30	I	*p*-NO_2_	20	<1
4	CuBr	2	30	I	H	10	5
5	none	0	30	I	H	10	0
6	none	0	3	I	H	10	0
7	2	2	0	I	H	10	0
8	2	0.5	3	I	*p*-OMe	40	70
9	2	0.5	3	I	H	40	85
10	2	0.5	3	I	*p*-CH_3_	40	52
11	2	0.3	3	I	*o*-OMe	40	53
12	2	0.3	3	I	H	40	36
13	2	0.3	3	I	*p*-CH_3_	40	25
14	2	0.3	3	I	*p*-OH	40	0
15	2	0.3	3	Br	*m*-Cl	40	4
16	2	0.3	3	Br	*m*-CH_3_	40	8
17	2	0.2	3	I	*p*-OMe	40	33
18	2	0.2	3	I	H^b^	40	43

^a^Functional group on the aryl halide. ^b^Reaction of naphthalene (2 g) with iodobenzene and 0.4 mL DMF.

Activation of the sp^2^ C–H bonds in naphthalene was possible as well. The reaction of iodobenzene in neat naphthalene at 85 °C using 0.2 mol % catalyst and 3 equivalents KO(*t*-Bu) gave a ≈2:1 ratio of 1-phenylnaphthalene and 2-phenylnaphthalene in a total yield of 43% (Table 1, entry 18). The observed substitution ratio is typical of substitutions on naphthalene under kinetic control that tend to favor the alpha C–H bonds due better resonance stabilization effects [53]. Importantly, we note the alpha protons are slightly more acidic than the beta protons [54].

The mechanism of Cu catalyzed coupling reactions and, more specifically, direct arylation have been the subject of intense interest. Mechanistic models appear to diverge along those favoring oxidative addition/reductive elimination via Cu(I)/Cu(III) versus proposals favoring a single electron transfer (SET) pathway [55–56].

In the base-promoted SET mechanism [57], electron donation to ArI leads to a short-lived radical anion ArI^•−^ that decomposes to I^−^ and Ar^•^. The Ar radical undergoes homolytic aromatic substitution with benzene to form a biaryl radical, and deprotonation gives a biaryl radical anion that transfers one electron to ArI to begin the cycle anew. The mechanism explains the metal-free direct arylation catalysis, but there has been some question about the initiation step. As KO(*t*-Bu) does not have sufficient reducing power to generate ArI^•−^, it has been proposed that initiation could arise from the reaction of ArI and KO(*t*-Bu) to form benzyne or that organic electron donors form in situ with suitable additives under the basic conditions of the reaction [58–60]. For example, observation of faster direct arylation in the presence of DMF led to proposals of deprotonated DMF as one electron donor [61] or forming a dibasic -enediol in situ as an electron donor [62].

**Scheme 2:**
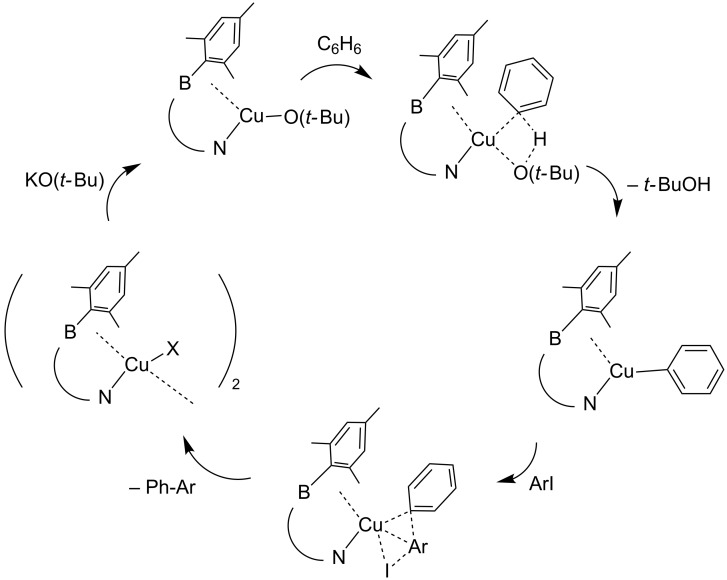
Proposed mechanism of direct arylation catalyzed by **2** (X = Cl/I; Ar = aryl).

While the SET mechanism has drawn much interest, our observation of large rate enhancement upon addition of the preformed catalyst **2** may be better described with a metalation–deprotonation step followed by oxidative addition/reductive elimination (Scheme 2) [63]. Thus, *t*-BuO^−^ substitutes the halogen ligand on **2**, followed by deprotonation–metalation of benzene, followed by oxidative addition of the arylhalide, and finally reductive elimination of the biaryl. The molecule **2** may be uniquely suited for this pathway. The electron-rich Cu(I) in the (L-Z)Cu(OR) intermediate may be well-stabilized by boron as a Z-type ligand, and the mesityl groups surrounding boron may favor the association of arenes for the deprotonation–metalation and the oxidative addition steps. Wang et al. proposed concerted metalation–deprotonation via a sigma bond metathesis involving a cyclic 4-membered transition state, followed by oxidative addition of ArI to Cu(I) [63]. Oxidative addition of arylhalide to Cu(I) produces Cu(III) intermediates, for which there is substantial evidence [64–67]. However, under catalytic conditions, there is no requirement that the copper catalyst pass through an intermediate with a formal oxidation state of +3, which may undoubtedly have a large activation energy. Rather, a concerted pathway through a 4-membered transition state will have less localization of charge. A concerted process for coupling of nucleophiles with arylhalides on copper centers have been proposed by Bacon [68] and were elaborated by Litvak [69] who proposed SET within the 4-membered transition state. It is noteworthy that modern DFT calculations [63] also produce cyclic transition states.

## Conclusion

In conclusion, we observe direct arylation reactions (C–X/C–H; X = halogen) catalyzed by a Cu(I) center stabilized by an ambiphilic ligand. The activation of stable sp^2^ C–H bonds in benzene and naphthalene occurs as a result of the catalysis. We favor a mechanism involving 4-membered cyclic transition states for metalation–deprotonation followed by concerted oxidative addition/reductive elimination. The scope of reactivity, including functional group tolerance on the reactants, types of C–H bonds that can be activated, selectivity of C–H bond activation, further optimization studies, and new catalyst design will be topics for further study. The initial results are very encouraging in that an inexpensive copper catalyst at low loading exhibited good activity for the reaction. The observation of high activity using a preformed copper catalyst may assist in the development of new catalysts.

## Experimental

**General**. Compounds **1** and **2** were prepared according to the literature [50]. All organic reagents and solvents were obtained from commercial sources and used without further purification. A GCMS-QP2010SE gas chromatograph–mass spectrometer (Shimadzu Corp., Kyoto, Japan) was used for GC–MS analyses. NMR spectra were recorded on an Avance 400 MHz spectrometer (Bruker, Billerica, MA, USA).

**Catalysis experiments**. A 50 mL roundbottom flask was charged with 0.5 mmol of aryl halide, benzene (4 mL) and 1.5 mmol of KO(*t-*Bu). The flask was fitted with a reflux condenser left open to air. Then, a solution of catalyst dissolved in 420 μL DMF was added to the reaction. The reaction was then stirred and refluxed for 40 h. The reaction was worked up by extraction with ether and washed with deionized H_2_O. The organic phase was collected and dried over anhydrous sodium sulfate. The residue was purified by flash column chromatography. NMR spectra of isolated products matched well with the literature.

## Supporting Information

File 1 NMR spectra and GC–MS data of the products.
