# Supplementary material for: Direct arylation catalysis with chloro[8-(dimesitylboryl)quinoline-κN]copper(I)
Source: Beilstein J Org Chem. 2016 Dec 15;12:2757–62. doi: 10.3762/bjoc.12.272 (PMC5238606; doi:10.3762/bjoc.12.272)
Supplement: File 1 — NMR spectra and GC–MS data of the products. [file Beilstein_J_Org_Chem-12-2757-s001.pdf]

Supporting Information

for

Direct arylation catalysis with chloro[8-(dimesitylboryl)quinoline-κN]copper(I)

Sem Raj Tamang and James D. Hoefelmeyer*

Address: Department of Chemistry, University of South Dakota, 414 E. Clark St., Vermillion, SD 57069, USA

Email: James D. Hoefelmeyer - jhoefelm@usd.edu

* Corresponding author

NMR spectra and GC–MS data of the products

Contents:

Figure S1: ^{1}H NMR of biphenyl. S3
Figure S2: ^{1}H NMR of 4-methoxybiphenyl. S4
Figure S3: ^{1}H NMR of 4-methylbiphenyl. S5
Figure S4: GC–MS data of biphenyl. *m/z* = 154. .... S6
Figure S5: GC–MS data of direct arylation at 1 and 2 position of naphthalene. *m/z* = 204. .... S7
Figure S6: GC–MS data for *p*-methoxybiphenyl. *m/z* = 234. .... S8
Figure S7: GC–MS data for *m*-chlorobiphenyl. *m/z* = 188 .... S9
Figure S8: GC–MS data for *p*-nitrobiphenyl. *m/z* = 199. .... S10
Figure S9: GC–MS data for *m*-methylbiphenyl. *m/z* = 168. .... S11

Experimental

General Conditions

Compound 1 and Chloro[8-(dimesitylboryl)quinoline-κN]copper(I) was prepared according to the literature [**1**]. All organic reagents and solvents were obtained from commercial sources and used without further purification. A GCMS-QP2010SE gas chromatograph-mass spectrometer (Shimadzu Corp., Kyoto, Japan) was used for GCMS analyses. NMR spectra were recorded on an Avance 400 MHz spectrometer (Bruker, Billerica, MA, USA).

Experimental Conditions

A 50 mL roundbottom flask was charged with 0.5 mmol of aryl halide, benzene (4mL) and 1.5 mmol of KO*t*Bu. The flask was fitted with a reflux condenser left open to air. Then, a solution of catalyst dissolved in 420µL DMF was added to the reaction. The reaction was then stirred and refluxed for 40 hrs. The reaction was worked up by extraction with ether and washing with DI H_2O. The organic phase was collected and dried over anhydrous sodium sulfate. The residue was purified by flash column chromatography. NMR spectra of isolated products matched well with the literature.

[**1**] Son, J.H.; Pudenz, M.A.; Hoefelmeyer, J.D. *Dalton Trans*. **2010**, *39*, 11081-11090.

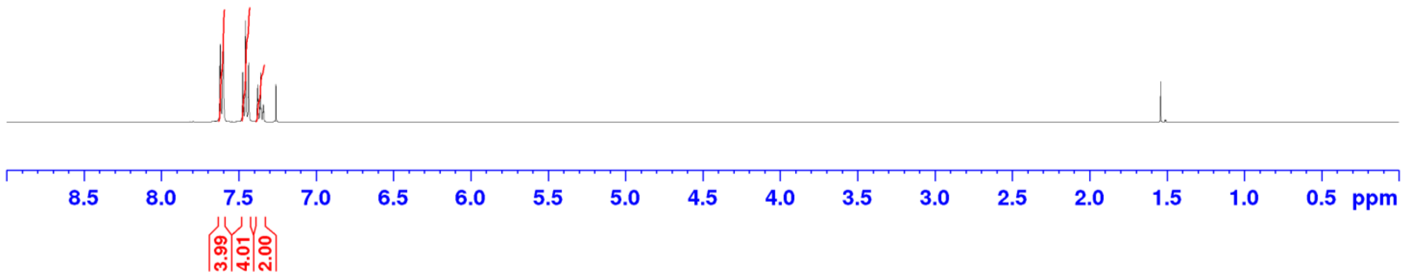

Figure S1: ^{1}H NMR of biphenyl in $CDCl_3$.

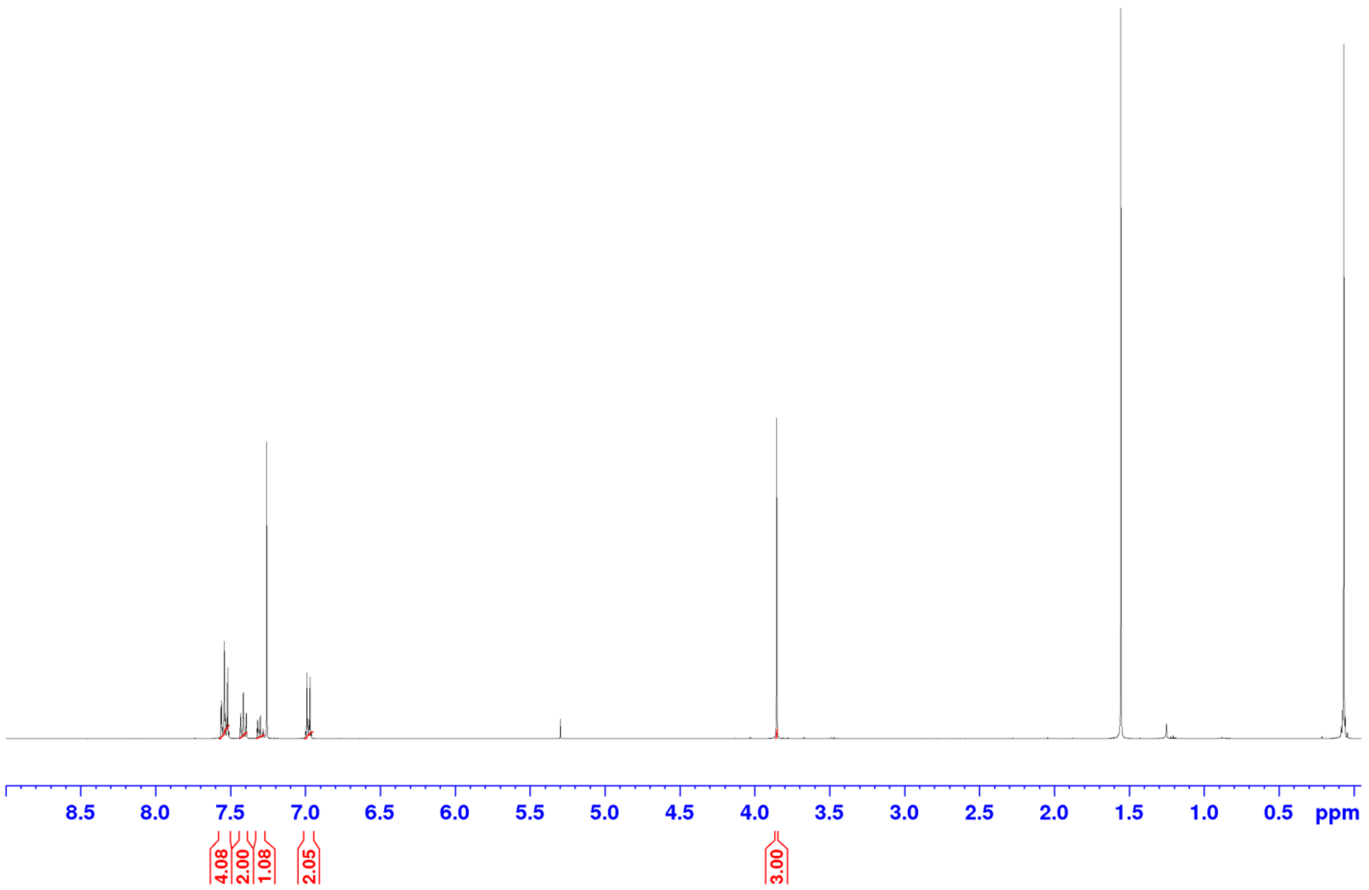

Figure S2: ^{1}H NMR of 4-methoxybiphenyl in $CDCl_3$.

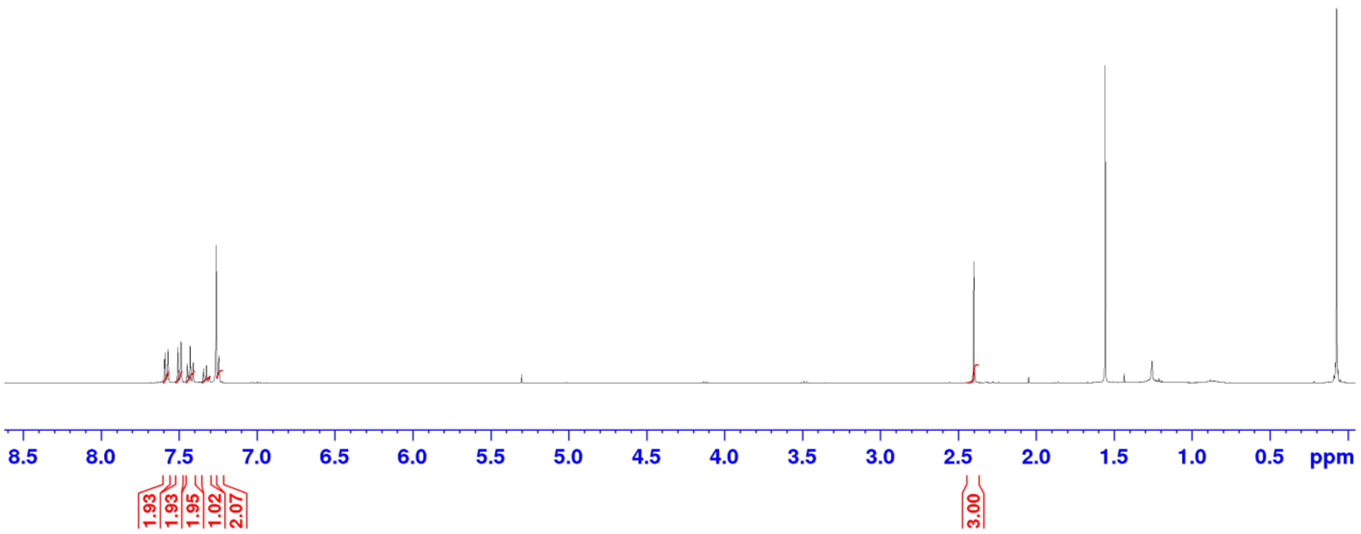

Figure S3: ^{1}H NMR of 4-methylbiphenyl in $CDCl_3$.

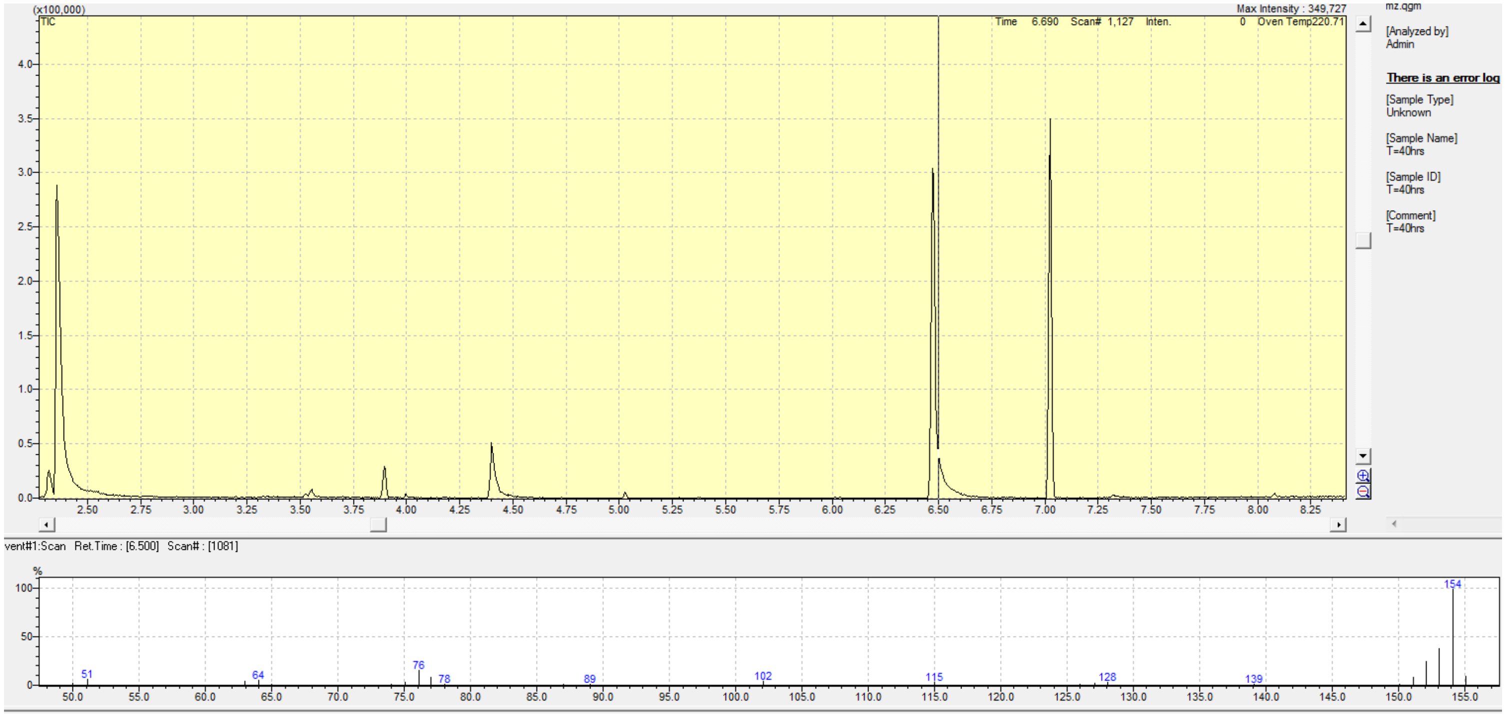

Figure S4: GC–MS data of biphenyl. m/z = 154.

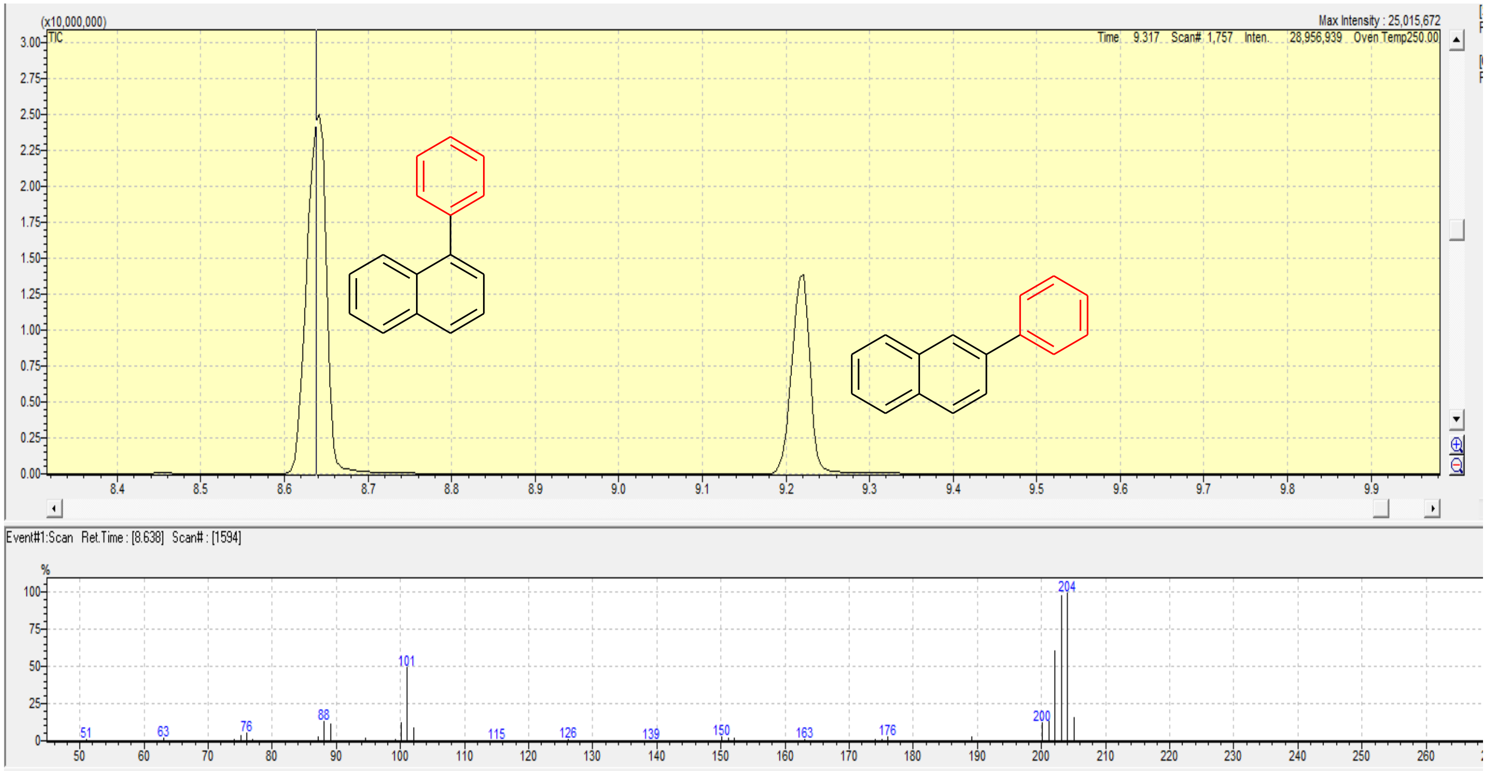

Figure S5: GC–MS data of direct arylation at 1 and 2 position of naphthalene. $m/z = 204$.

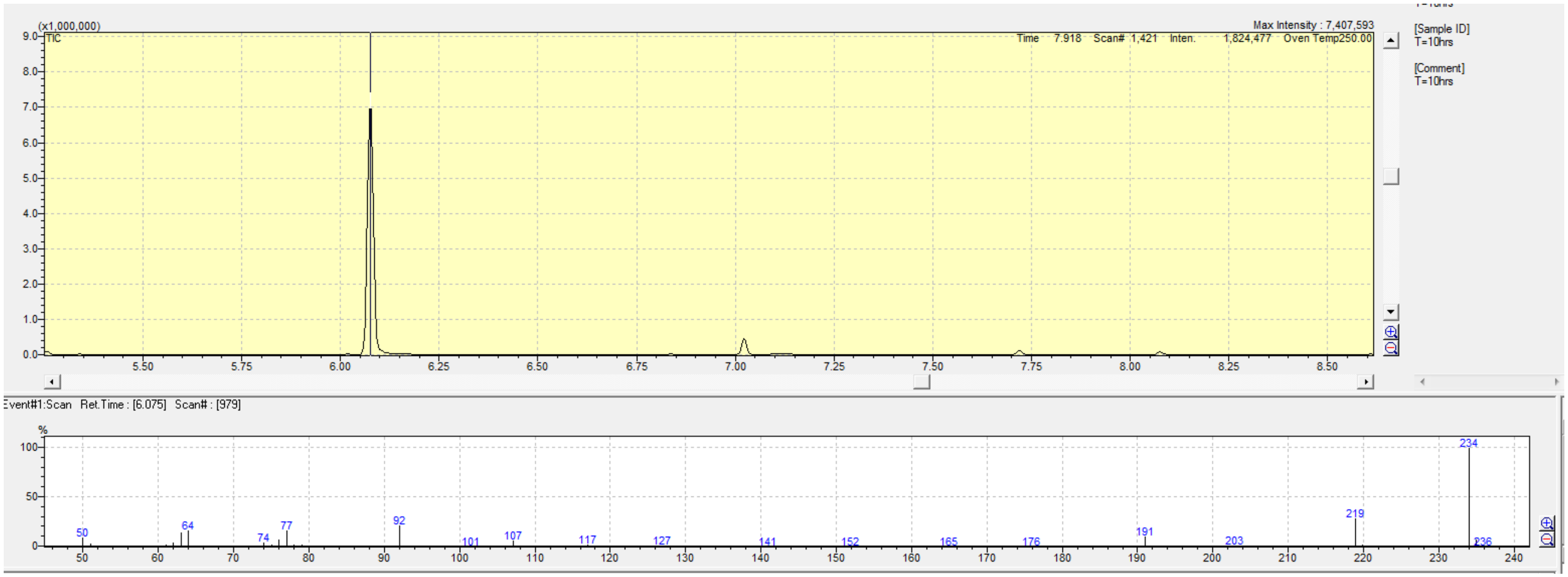

Figure S6: GC–MS data for *p*-methoxybiphenyl. *m*/*z* = 234.

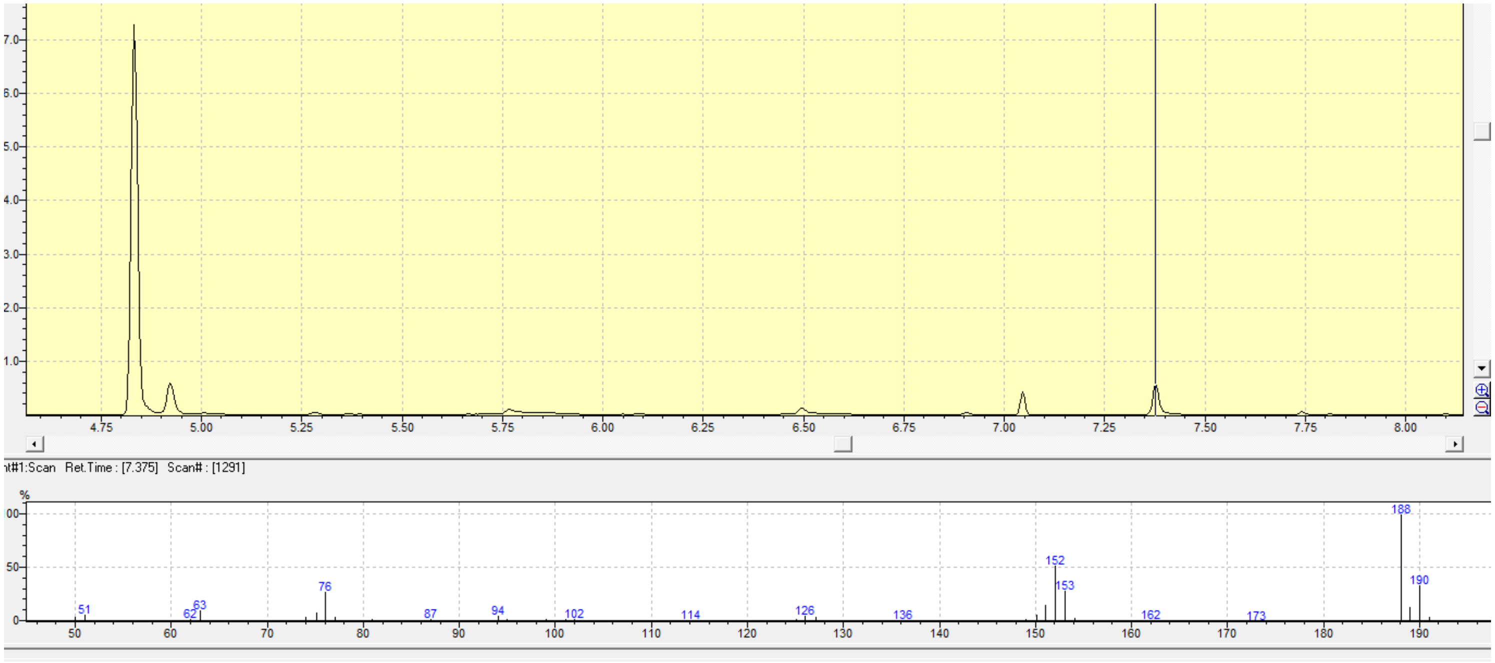

Figure S7: GC–MS data for *m*-chlorobiphenyl. m/z = 188.

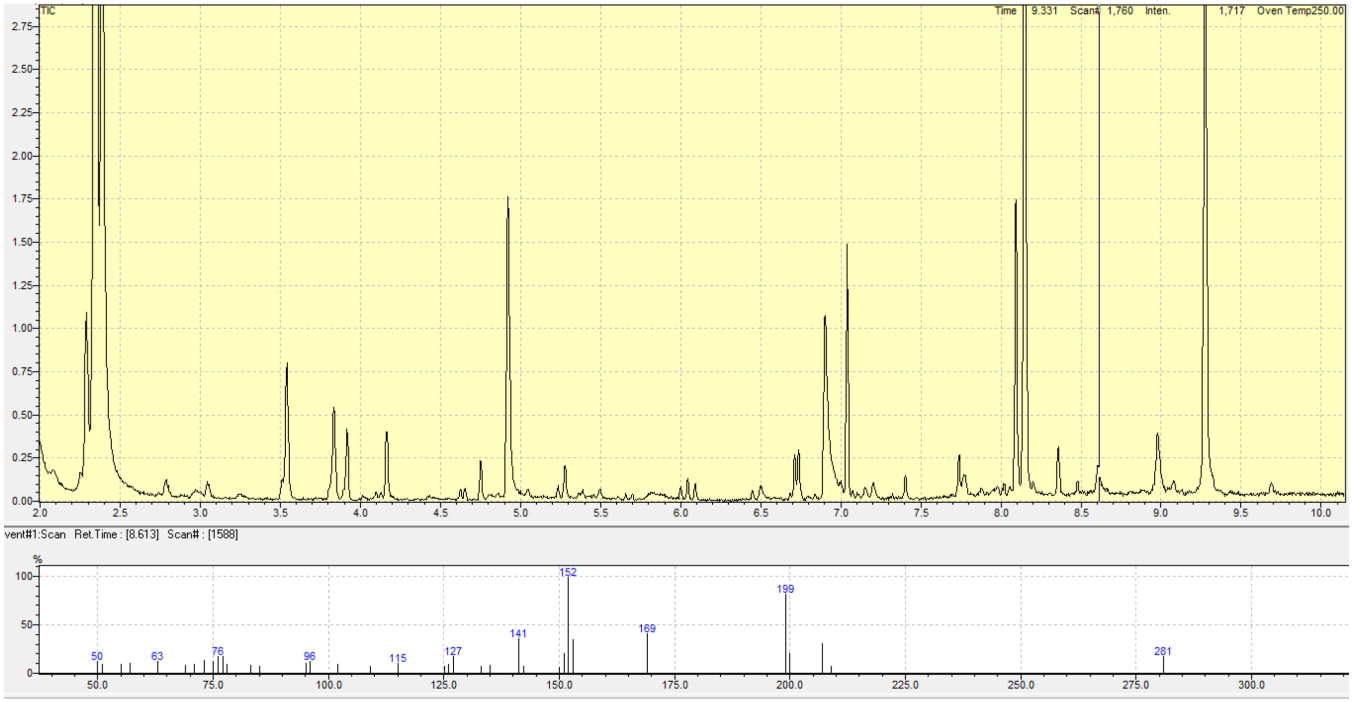

Figure S8: GC–MS data for *p*-nitrobiphenyl. *m*/*z* = 199.

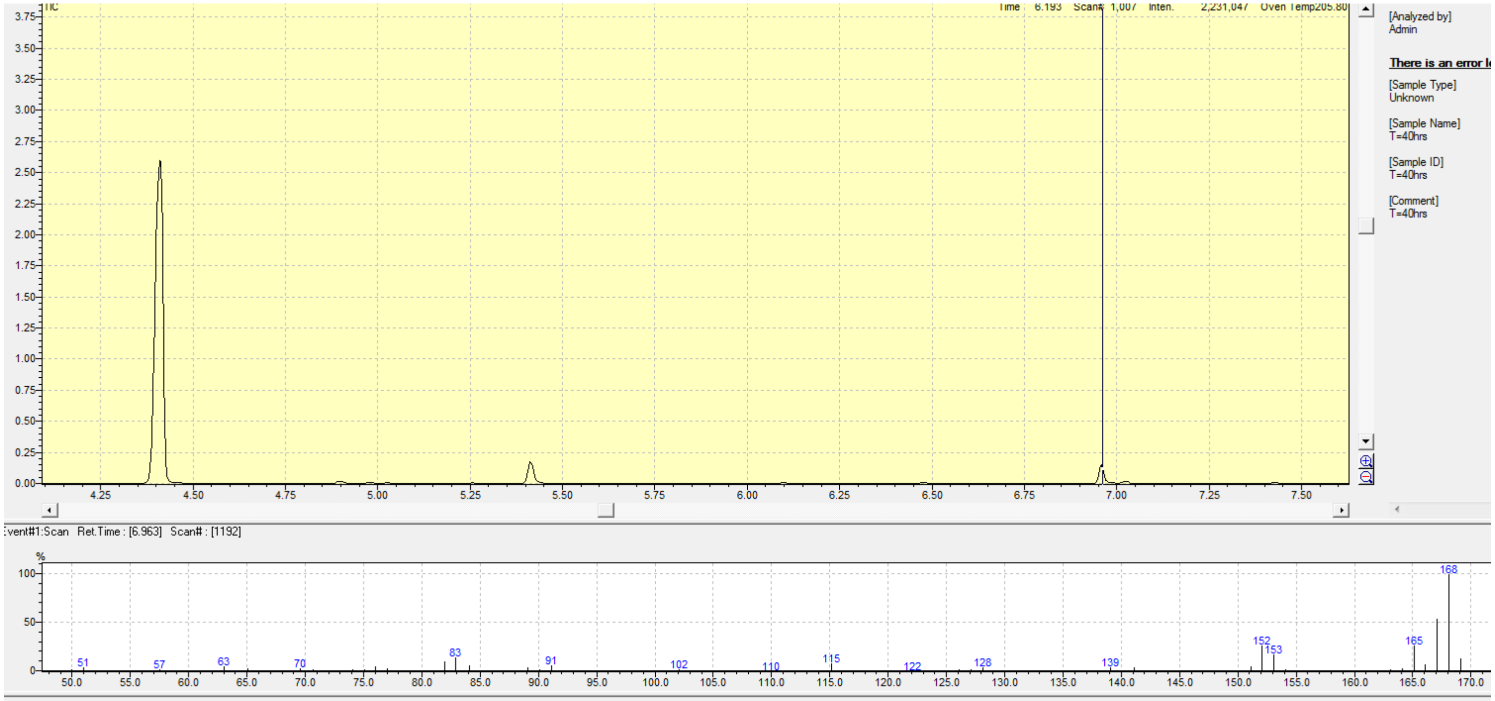

Figure S9: GC–MS data for *m*-methylbiphenyl. m/z = 168.
